# Overexpression of Capsular Polysaccharide Biosynthesis Protein in *Lactobacillus plantarum* P1 to Enhance Capsular Polysaccharide Production for Di-n-butyl Phthalate Adsorption

**DOI:** 10.4014/jmb.2101.01026

**Published:** 2021-04-08

**Authors:** Wei-Bing Liu, Zhi-Wei Lin, Ying Zhou, Bang-Ce Ye

**Affiliations:** Lab of Biosystems and Microanalysis, Biomedical Nanotechnology Center, State Key Laboratory of Bioreactor Engineering, East China University of Science and Technology, Shanghai 200237, P.R. China

**Keywords:** *Lactobacillus plantarum*, exopolysaccharides, probiotics, overexpression, di-n-butyl phthalate, adsorption

## Abstract

Exopolysaccharides (EPSs) such as capsular polysaccharide (CPS) are important bioactive carbohydrate compounds and are often used as bioenrichment agents and bioabsorbers to remove environmental pollutants like di-n-butyl phthalate (DBP). Among the EPS-producing bacteria, lactic acid bacteria (LAB) have gained the most attention. As generally recognized as safe (GRAS) microorganisms, LAB can produce EPSs having many different structures and no health risks. However, EPS production by LAB does not meet the needs of large-scale application on an industrial scale. Here, the *capA* gene (encoding CPS biosynthesis protein) was overexpressed in *Lactobacillus plantarum* P1 to improve the production of EPSs and further enhance the DBP adsorption capability. Compared with P1, the CPS production in *capA* overexpressed strain was increased by 11.3 mg/l, and the EPS thickness was increased from 0.0786 ± 0.0224 μm in P1 to 0.1160 ± 0.0480 μm in P1-*capA*. These increases caused the DBP adsorption ratio of P1-*capA* to be doubled. Overall, the findings in this study provide a safe method for the adsorption and removal of DBP.

## Introduction

Exopolysaccharide (EPSs) are known as carbohydrate polymers with high molecular weight, and are composed of monosaccharide residues. Generally, EPSs are in the cell wall through extracellular in situ synthesis or secreted into the extracellular environment and play various vital roles due to their biocompatibility and non-toxicity. EPSs are often used as bioenrichment agents, bioabsorbers, heavy metal removers, and drug delivery agents in biology and medicine [1]. In addition, EPSs also possess excellent viscosity and rheology and have been explored for application in the dairy industry. So far, many studies have shown that EPSs can be produced by plants, algae, and fungi as well as bacteria [2]. Among bacteria, lactic acid bacteria (LAB) have been widely used to produce EPSs to prolong the shelf-life of fermentation food and retain its flavor because of the ability of EPSs in impacting viscosity, syneresis and sensory properties [3-5]. LAB are generally recognized as safe (GRAS) microorganisms in food. Therefore, LAB are well suited for producing EPSs for adsorbing and removing environmental pollutants, and several previous works have demonstrated that LAB can eliminate tenuazonic acid, acrylamide, or mycotoxin in vivo or in vitro [6-8]. However, the EPS production of LAB does not meet the needs large-scale application in industry. The rapid development of molecular biological technology has served to elucidate the biosynthesis pathway of EPSs. The use of genetic engineering methods is an important way to increase the production of EPSs in lactobacillus. In addition, researchers have analyzed the enzymes related to EPS production in lactobacillus and found that the activities of UDP-galactose-4-epimerase, α-phosphoglucomutase and UDP-glucose pyrophosphorylase are important in the cell extract [9]. Boels *et al*. overexpressed the whole gene cluster related to the synthesis of EPSs in *Lactococcus*, which could enhance the production of EPSs by four times to 345 mg/l. Boels *et al*. modified *Lactococcus lactis* by metabolic engineering to alter the carbon distribution between glycolysis and nucleotide sugar biosynthesis, thus increasing the intracellular levels of UDP-glucose, UDP-galactose and UDP-rhamnosus, and increasing EPS production [10].

**Fig. 1:**
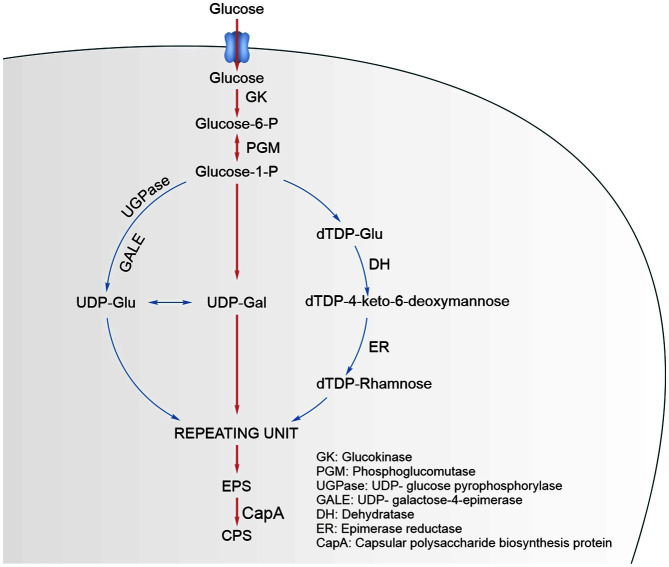
The biosynthetic pathway of CPS.

The synthesis of EPSs is a quite complex biological process, involving a variety of enzymes and regulatory proteins [11, 12]. It contains four major steps: sugar transportation, synthesis of sugar-1p, polymerization of repeating units, and EPS exportation [13]. Some EPSs can be categorized as capsular polysaccharides (CPS), which are covalently bound to the outer cell membrane (Fig. 1). Another type are called slime polysaccharides (SPS), which are released from the cell wall [14, 15]. CPS biosynthesis protein (CapA) is required for the biosynthesis of type I CPS [16]. This protein is involved in the biosynthesis pathway of CPS, and is concerned with capsule biogenesis and is essential for high-level polymerization. Beyond these works, there have been few studies on the CPS production of LAB, therefore, research on increasing the yield of CPS will be helpful for the application of LAB in the absorption and removal of toxins such as di-n-butyl phthalate (DBP from the human body).

DBP is widely used in many daily plastic products as a plasticizer for packaging material for food, personal toiletries, and plastic toys [17]. Furthermore, evidence has shown that DBP can damage the genital system and behavior of human beings and wildlife with long-term exposure, and even at very low dose [18-20]. Within natural the environment, the removal of DBP mainly relies on microbial adsorption and degradation [21].

In a previous work, we studied the feasibility of DBP adsorption by LAB [22]. Here, we aimed to increase the CPS production of *L. plantarum* P1, and explored how to enhance the function of DBP adsorption in LAB. First, the vector pMG36e [23] is an effective tool for genetic engineering of LAB. The ability whereby pMG36e constitutively expresses an inserted gene in LAB indicated that it could be modified to generate the novel food-grade expression vector based on it [24]. Many studies have proved that the vector pMG36e is an effective expression vector for genetic engineering of LAB [25-30], so the lactobacillus universal plasmid pMG36e is used to overexpress the CPS biosynthesis protein (CapA), the endogenous gene of *L. plantarum* P1. In addition, some previous works demonstrated that LAB could bind to DBP [31, 32]. In our study we investigated the effects of *capA* overexpression on DBP adsorption. This work may help extend the application of LAB in eliminating DBP from the environment.

## Materials and Methods

### Bacterial Culture and Plasmid Purification

The strains and plasmids used in this study are listed in Table 1. *L. plantarum* P1 was cultured in MRS broth (Solarbio Co., China) at 30°C for 24 h. Chemically competent *Escherichia coli* (*E. coli*) DH5α (TransGen Biotech, China) was used for recombinant construction. Cells were cultivated on 90 mm disks with Luria-Bertani (LB) medium at 37°C. Erythromycin was used as a selection marker during cloning. For the selection of erythromycin-resistant *E. coli* DH5α and *L. plantarum* P1 strains after transformation, either 600 μg/ml or 2.5 μg/ml of erythromycin was used. Plasmids in *E. coli* were purified with an EasyPure Plasmid MiniPrep Kit (Beijing TransGen Biotech Co., Ltd.) according to the instructions with the kit.

### Construction of the Recombinant Plasmid pMG36e-Flag-*capA*

Polymerase chain reactions (PCR) were performed in a PCR thermal cycler (Bio-Rad, USA) with 50 μl of reaction mixture containing the template (1 μl), each primer (2 μl), polymerase (1 μl), dNTP (1 μl), deionized water (18 μl), and 10× buffer (25 μl). The PCR was run following this program: pre-denaturation at 95°C for 5 min, followed by 30 cycles of denaturation at 95°C for 15 sec; annealing at 57°C for 1 min and extension at 72°C for 1min.

The *flag-capA* gene was cloned by PCR using specific primers *capA*-F1/*capA*-R1 (*capA*-F1:5'-AAGGACGAC GATGACAAGGATCAAGCAGTAAGTTTTGACTTTTTTATCCGG-3' and *capA*-R1:5'-TTCAGACTTTGC AAGCTTCTAAACTCGTTTCATCGCTTGTGACG-3') (Table 1). The PCR fragment containing *flag-capA* gene was cloned into vector pMG36e to generate new plasmid pMG36e-*flag-capA*. The new resultant plasmid was transformed into *L. plantarum* P1 by electroporation at 1.25 kV/cm in a Bio-Rad GenePulser, and then the cells were diluted with 400 µl of regeneration medium MRS. The competent cells of *L. plantarum* P1 were prepared following the protocol below. *L. plantarum* P1 cells were cultured in MRS at 30°C overnight. A total of 50 mL MRS with 1% glycine and 0.75 M sorbitol was inoculated with 1% of the overnight culture. At OD_600_ of 0.3, the cells were harvested by centrifugation at 6,000 ×*g*, and then washed by sterile water for three times and stored in 1/125 30%PEG3000 [33].

### Reverse Transcription Quantitative Real-Time PCR Analysis

Total RNA from recombinant *L. plantarum* P1 strains was isolated and purified by using the RNA Prep Pure Cell/Bacteria Kit (Tiangen, China). The completeness of the RNA was verified by 1.0% of agarose gel electrophoresis with a microplate reader. Total RNA (1 μg) was reversely transcribed using the PrimeScript RT Reagent Kit with gDNA Eraser (TaKaRa, Japan). Genomic DNA was removed by digestion with DNase for 5 min at 42°C. RT-qPCR was performed using a SYBR Premix Ex Taq GC kit with a template cDNA of 75 ng in a CFX96 real-time PCR instrument. Primers listed in Table 1(*capA*-F2/*capA*-R2) were used. The reaction conditions were: 95°C, 10 min, then 95°C, 10 s, 60°C, 10 s for 39 cycles, and finally 72°C, 30 s. The internal reference gene is 16S rRNA. All samples were prepared in triplicate to obtain the C_T_ values, and the relative gene expression levels for each mutant (compared with WT levels) were calculated using the comparative C_T_ method (2^−ΔΔCt^) [34].

### Western Blot Analysis

The P1 mutants were cultured in MRS medium containing erythromycin for 24 h to make the protein fully expressed. After lysozyme treatment, it was sonicated to obtain the crude enzyme of extracellular polysaccharide synthesis protein.

*L. plantarum* P1 cell pellets were resuspended in phosphate-buffered saline and subjected to ultrasound breaking, then they were diluted in 6× protein loading buffer (Trans Biotech, China) and heated for 5 min. Samples were run on a 10% sodium dodecyl sulfate polyacrylamide gel at 110V in Tris-glycine buffers. Gels were then transferred to polyvinylidene difluoride membranes which were incubated with primary antibodies in TBS-T (TBS 0.1% Tween 20) for 12 h at 4°C. Next, the membranes were washed three times in TBS-T and incubated for 1 h with antigoat IgG conjugated with horseradish peroxidase (HRP, Cell Signaling) at a 1/5000 dilution in TBS-T. Finally, the membranes were washed three times in TBS-T and the HRP signal was detected.

### Growth Analysis and CPS Production

*L. plantarum* P1 and its mutants were grown in MRS at 30°C, and in triplicate 50 ml cultures. Growth was analyzed using a microplate reader (BioTek Instruments, USA). Cell density measurements at OD_600_ were acquired every 4 h. To evaluate CPS production, phenol-sulfuric acid method was used [35]. The bacterial solution was obtained through activation culture, and the cells were obtained by centrifugation. The cells were washed once with physiological saline. Then, the cells were suspended in a glycine buffer (0.1 M, pH 9.2), and 100 mg of lysozyme and 0.02% of sodium azide was added to inhibit the growth of bacteria, followed by shaking at 37°C for 7 h and centrifuging (8,000 rpm, 10 min) to remove the bacterial residue precipitation. 0.1 M CaCl_2_ was added to the supernatant, shaking for 1 h, and then adding 25% (v/v) of absolute ethanol. The samples were placed at 4°C for 2 h and centrifuged (8,000 ×*g*, 10 min) to remove the precipitate, and absolute ethanol was added to a final concentration of 80% (v/v). The samples were then left at 4°C overnight to precipitate, and centrifuged to obtain crude CPS.

To evaluate the CPS titer, a standard curve was drawn with the value of OD_490_ of glucose as ordinate and the value of concentration (g/ml) as abscissa. The OD_490_ and concentration of glucose have a good linear relationship; the linear regression equation is y = 0.0097x + 0.0689 (R^2^ = 0.99845), and the changes of CPS content in the two strains after culturing for 64 h were calculated according to this equation.

### Observation of EPSs

The CPS bound to the cell wall, which could be observed by transmission electron microscopy [36]. So, the activated culture was centrifuged at 4,000 ×*g* for 10 min, washed twice with PBS (pH 7.4), suspended in PBS (pH 7.4), and diluted to an OD_600_ of 0.3-0.4. Then, 20 μl of that was dropped on a copper mesh. This was left for 20 min the droplets were sucked up with filter paper, and then the copper mesh was placed on a transmission electron microscope for observation.

### DBP Adsorption Evaluation

To evaluate the DBP adsorption capacity of *L. plantarum* P1 and its mutants, we tested the DBP (>99.0%, D&B Chemical Technology Co. Ltd., China) adsorption capacity of the strains. Two strains were inoculated from cryopreserved stocks and incubated at 30°C for 24 h. The cultures were harvested by centrifugation (8,000 ×*g*, 10 min, 4°C), washed twice and resuspended to achieve a density of 2 × 10^11^ colony-forming units (CFU)/ml using either normal saline (0.9% NaCl w/v) or DBP solution (10 mg/ml). The samples were incubated at 37°C for 2 h without shaking. After incubation, the suspension was centrifuged (4,000 ×*g*, 5 min) at room temperature, and the supernatant was extracted by ethyl acetate for the analysis of residual DBP contents. After the extraction, 200 µl of supernatant was transferred to a 5-ml glass bottle, and then washed with 1 ml of methyl alcohol. The eluent was collected and filtered through a 0.22-µm membrane filter, and this was followed by high-performance liquid chromatography (HPLC) analysis to measure unbound DBP. The chromatographic separation was performed on a C_18_ column (250 × 4.6 mm I.D., 5 μm; Teknokroma, Spain). The mobile phase for detecting DBP was methanol: water (80:20, v/v), the flow rate was 1 ml/min, and UV detection was done at 225 nm. All the assays were conducted in triplicate, and average values were used for data analysis. The adsorption rate of DBP was calculated as follows: adsorption rate = (10-C1) /10×100% (C1: the concentration of DBP in methanol).

## Results

### Construction of the Vector pMG36e-Flag-*capA*

**Fig. 2:**
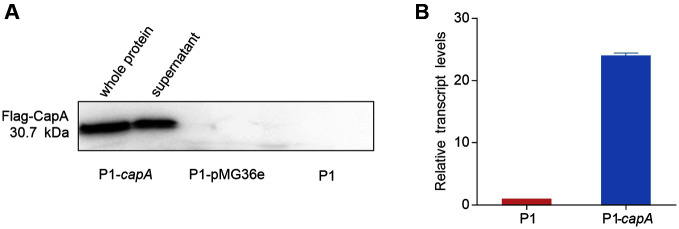
Validation of *capA* overexpression. (**A**) Western blot analysis of CapA in recombinant bacteria; P1 without the plasmid and P1 with the original plasmid pMG36e were used as controls. (**B**) Analysis of *capA* gene overexpression at transcriptional level.

To overexpress *capA* gene, the plasmid pMG36e-*flag-capA* was constructed and transformed into P1 strain, which was then validated by 16S rRNA gene sequecning. The recombinant strain was treated by lysozyme and sonication for extracting extracellular polysaccharide synthesis protein, in which, supernatant was collected for western blot characterization. In silico analysis indicated that the protein size of CapA is 30.7 kDa with 256 amino acids. As shown in Fig. 2A, positive bands of CapA were observed in whole protein and supernatant samples, while no target proteins were found in P1-pMG36e and P1, respectively. This data demonstrated that *capA* was overexpressed successfully in P1.

To evaluate the expression level of *capA* in P1-*capA*, RT-qPCR assay was performed with 16S rRNA gene as an internal reference gene. As shown in Fig. 2B, the expression of *capA* in P1-*capA* is significantly higher than that in P1. The result showed that the recombinant strain promotes the expression of *capA*, which will increase the production of CPS.

### CPS Yield Evaluation

**Fig. 3:**
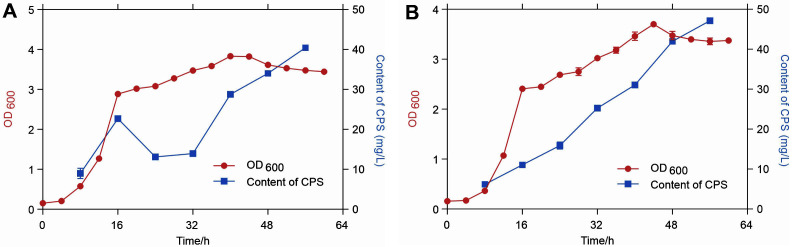
Evaluation of strain growth and CPS production in P1 (**A**) and P1-*capA* (**B**).

To analyze the correlation of growth of P1 and P1-*capA* strains and the production of CPS in them, the growth curve at OD_600_ with CPS production was tested. As shown in Fig. 3A, the production of CPS has an obvious oscillation from 24h to 32h in P1 with growth time continuing. The production of CPS in P1-*capA* continuously increased (Fig. 3B). After 24 h cultivation, the yield of CPS of P1-*capA* was higher than that of P1, especially at the time point of 32 h. The CPS production in P1-*capA* was higher than that in P1 by 11.3 mg/l. Therefore, the growth time of 32 h was selected as the reference time point for further studies.

### Morphological Change of Strain

**Fig. 4:**
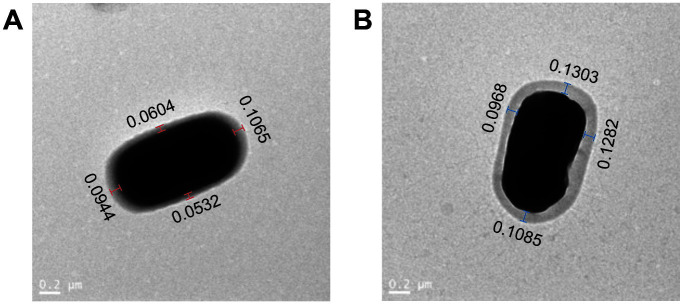
Transmission electron microscopy for illustration of EPSs in P1 (**A**) and P1-*capA* (**B**). The numbers represent the thickness (micrometer) of capsular polysaccharide in P1 (**A**) and P1-*capA* (**B**).

Transmission electron microscopy (TEM) was used to observe the changes and differences of CPS in P1 and P1-*capA*. The two strains were collected for TEM observation after 32 h of cultivation. As seen both in P1 (Fig. 4A) and P1-*capA* (Fig. 4B), the EPS thicknesses of P1 and P1-*capA* were in the range of 0.0786 ± 0.0224 and 0.1160 ± 0.0480 µm respectively. Although the EPSs were uneven around the cell, the EPS thickness of P1-*capA* was significantly higher than that of P1 in each of the same locations. This result indicated that the production of EPSs increased after *capA* overexpression in P1-*capA*.

### Comparison of DBP Adsorption Capacity

**Fig. 5:**
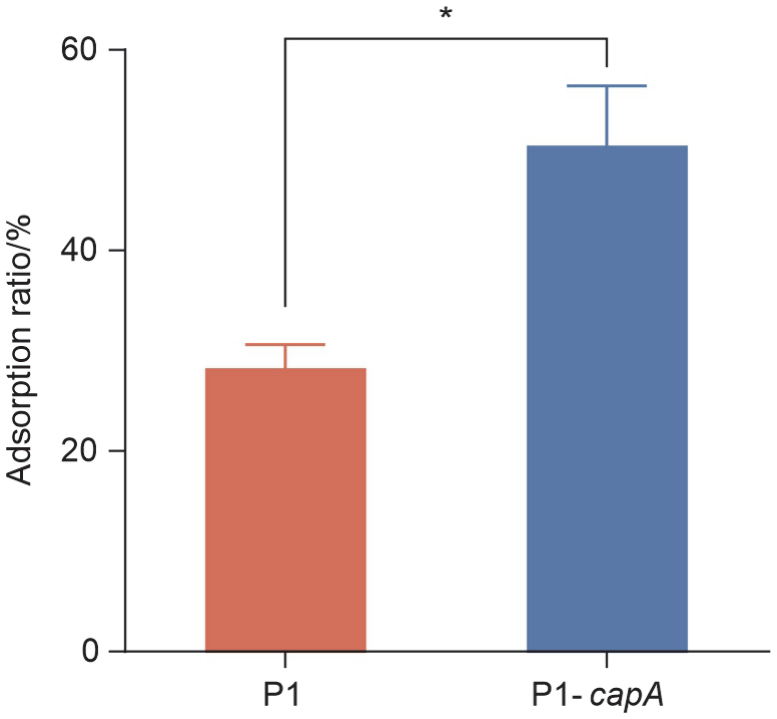
DBP adsorption capacity of P1 and P1-*capA*. The experiments were duplicated three times and the data were presented with standard deviation. *p < 0.05.

LAB as a GRAS microorganism is widely used to adsorb and remove harmful substances such as zearalenone, acrylamide and heavy metals [37-39]. Here, we compared the DBP adsorption ratio of P1 and P1-*capA* after 32 h of cultivation. As shown in Fig. 5, the adsorption ratio of DBP in P1-*capA* with the overexpression of CapA protein was significantly higher than that in P1. It displayed that the CPS in both P1 and P1-*capA* could play roles in DBP adsorption, and the overexpression of CPS significantly enhanced the adsorption ratio to 50.48%.

## Discussion

EPSs are one of the main components of bacterial cell wall, and the biosynthesis of it can be affected by many factors from the genetic structure of bacteria to cultural conditions [40, 41]. To increase the production of EPSs, researchers have investigated various pathways to achieve this goal. For example, Mıdık *et al*. recently discussed the effect of different culture conditions (including temperature, pH, and incubation time) on EPS production in lactic acid bacteria [42]. So far, the highest production of EPS in lactobacilli was reported as 2.775 g/l in *L. rhamnosus* RW-9595 M [43], and the second highest production was 2.5 g/l in *L. kefiranofaciens* WT-2B [44]. Here, we obtained 11.3 mg/l higher EPS production compared with the wild-type strain by overexpressing *capA* gene in *L. plantarum* P1. This provides a new strategy to enhance EPS production in LAB, which makes it possible for LAB strains to be used for the removal of toxic substances, such as DBP. In addition, we will further increase EPS production in LAB by screening starting strains, and optimizing culture conditions and fermentation parameters in the future.

In recent years, many studies demonstrated that LAB strains were widely used to adsorb and eliminate harmful substances [45]. In previous work, we have evaluated the adsorption of DBP by LAB, and the data indicated that the P1 strain could effectively adsorb DBP [22]. In this study, we constructed an overexpression LAB strain, which indicated that CPS thickness of P1-*capA* was increased to 0.13 µm. Therefore, the absorption efficiency of DBP in P1-*capA* was significantly higher by 50.48% than that in P1. As comparison, a previous study indicated that the DBP absorption in *Leuconostoc mesenteroides* DM1-2 was 45.00% [46]. In addition, the microbial elimination of DBP from the natural environment is the main goal and methodology. So far, many bacteria species were used to remove DBP from environment, in activated sludge [47, 48], soil [49, 50], and water [51, 52]. These bacteria can efficiently remove DBP from the environment in vitro, but are not suitable for usage in vivo. Compared with those bacteria, LAB is safe for human beings and has more potential for DBP adsorption and removal in vivo. Furthermore, except for our previous works [22], Zhao *et al*., also engaged in the study of DBP removal using LAB from the point of chemical binding [53]. In the present study, we applied overexpression strategy using *capA* gene to enhance the production of CPS, and significantly increased the adsorption efficiency. This study provides a safe method for the adsorption and removal of DBP and further provides insight for the low-cost removal of environmental pollutants.

## Figures and Tables

**Table 1:** Bacterial strains, plasmids and primers used in this study.

Strains and plasmids	Resource
*Escherichia coli* DH5α	Purchased from TransGen Biotech (Beijing) Co., Ltd.
*L. plantarum* P1	lab stock (isolated from Chinese traditional fermented food Naipizi)
pMG36e	lab stock
pMG36e-flag-*capA*	This study
*capA*-F1	This study
*capA*-R1	This study
*capA*-F2	This study
*capA*-R2	This study
